# Cancer-related fatigue in post-treatment cancer survivors: application of the common sense model of illness representations

**DOI:** 10.1186/s12885-016-2907-8

**Published:** 2016-11-25

**Authors:** Teresa Corbett, AnnMarie Groarke, Jane C. Walsh, Brian E. McGuire

**Affiliations:** School of Psychology, National University of Ireland Galway, Galway, Ireland

## Abstract

**Background:**

Cancer-related fatigue (CrF) is a common and disruptive symptom that may be experienced during and after cancer. Research into the subjective experience of fatigue in this group is required. The common sense model of self-regulation of health and illness (SRM) addresses personal beliefs or mental representations—whether medically sound or unsubstantiated— that a person holds about a health issue. The current study assesses if the SRM could be used as a theoretical framework for organizing the experiences of people with CrF, with a view to identifying methods to address fatigue in cancer survivors.

**Method:**

Four focus groups were held with a total of 18 cancer survivors who reported they experienced ‘significant fatigue or reduced energy.’ A thematic analysis was conducted within the framework of the SRM.

**Results:**

Findings were aligned with the SRM, with participants discussing fatigue with reference to representation, coping, and appraisal of symptoms. In particular, the wider social context of CrF was frequently addressed. Perceived inadequacies in support available to those with lingering fatigue after the completion of cancer treatment were highlighted by the participants.

**Conclusion:**

This study explored the subjective experience of fatigue after cancer using the SRM. CrF should be approached as a complex psychosocial issue and considered from the patient perspective to facilitate better understanding and management of symptoms. The SRM is an applicable framework for identifying modifiable factors that could lead to improved coping with CrF in post-treatment cancer survivors.

## Background

Up to 75 % of post-treatment cancer survivors experience negative health-related consequences [1]. The exploration of late effects of treatment, ongoing symptoms, survivorship care and self-management is now considered a priority [2]. Cancer survivors have specific emotional and physical needs [3], and research into these areas requires input from survivors [4].

Cancer-related fatigue (CrF) is the most common and disruptive symptom experienced by cancer survivors. It is a distressing, persistent, subjective feeling of physical, emotional and/or cognitive tiredness associated with cancer or cancer treatment [5]. CrF is more severe, enduring, and debilitating than “normal” fatigue caused by lack of sleep or overexertion and it is not relieved by sleep or rest [6]. Fatigue during treatment is a risk factor for developing chronic CrF following treatment [7]. Up to 30 % of cancer survivors experience persistent fatigue for years after cancer diagnosis [8]. It is an often un-treated symptom that contributes to diminished functioning, reduced quality of life, and socioeconomic consequences [9, 10]. Recently is an increased focus on the needs associated with treatment-induced symptoms of post-treatment cancer survivors [11]. These persistent negative effects delay the patients’ return to normal life [12].

Fatigue is often described as a medically-contested illness [13]. Individuals with fatigue report that a ‘medicalised’ self-identity is unavailable to them, in contrast to those impaired due to medically- and socially- legitimated illnesses [14]. The ‘invisible’ nature of fatigue may lead others to discredit patients’ illness experiences [15] and those with CrF have described a lack of understanding from family, friends and health professionals [16]. Consequently, they are often left to make sense of and manage CrF by themselves. A greater understanding of patient beliefs about their fatigue would be useful given evidence that certain types of thoughts (e.g., catastrophising) are associated with CrF [17].

The underlying aetiology of CrF is not well understood [18] but it is thought to be a complex process associated with physical, mental, and emotional aspects. Minton et al [19] note that the processes that cause and maintain fatigue overtime remain unclear. An inflammatory response to both the cancer itself and the range of treatment modalities has been linked to fatigue. [20]. Given that those who are post-treatment would generally be expected to improve overtime (when disease and treatment side effects had abated), it is hypothesised that other factors may lead to prolonged fatigue during survivorship. A cognitive-behavioural model of CrF posits that biological insults such as cancer or its treatment may precipitate the initial experience of fatigue during cancer, but behavioural and cognitive factors may aggravate and prolong fatigue in survivorship [17]. Thus the aims of this study are to explore the experience of fatigue in those after treatment rather than discussing the cancer experience or trajectory. In some instances, experiences particular to the individuals’ cancer experience were mentioned. However as outlined in the interview schedule, unless the comments referred directly to fatigue after cancer, these were not the focus of the study (see Appendix 2).

Guidelines for the support of individuals with CrF following treatment recommend the use of cognitive-behavioural therapy (CBT) [21, 22]. CBT aims to influence or change cognitions, emotions, behaviours, or a combination of these [23]. Interventions which target these processes may improve symptom management in cancer-related fatigue [24]. These interventions target knowledge, emotional adjustment, quality of life, coping skills, physical health and functional adjustment [25].

CBT interventions focus on similar cognitive, emotional and coping/behavioural factors as those outlined by Leventhal’s [26, 27] common sense model of self-regulation (or self-regulation model: SRM). This theoretical framework may, therefore, provide a useful approach to understanding post-treatment CrF with potential for informing the design of interventions based on cognitive–behavioural principles.

The SRM suggests that illness information - whether medically sound or unsubstantiated - is evaluated and integrated by the individual to provide a ‘lay’ understanding of the symptom or illness. Illness representations may be guided by current and prior awareness of symptoms, or by social messages from perceived significant others or authoritative sources [28]. Processing of information occurs in three stages: representation, coping, and appraisal [14].

The individual’s representation of illness is proposed to have 5 components [27]: identity (the name or label applied to the symptoms), timeline (the perceived time trajectory for the symptoms), consequences (expected future effects and outcomes due to symptoms), causes (beliefs about aetiology of the symptoms) and control (the extent to which the patient believes that they can gain personal control over the symptoms). Coping is guided by illness representations [29] and involves implementing responses for managing the symptoms or the emotional responses that follow. Viewing illness and symptoms as controllable is linked to active coping, whereas perceptions that symptoms are uncontrollable and chronic have been found to be associated with avoidance and denial coping [30]. An individual also appraises the effectiveness of their coping efforts [31] and this evaluation may result in a change in coping strategy and/or a change in perception of the illness and its symptoms [32].

The model has proved useful across many health conditions [29] with considerable evidence linking elements of the SRM to psychological functioning in a wide range of illnesses [30, 32, 33]. Few studies, report on how survivors describe CrF, highlighting the need for research from the patient perspective [15] and only one study to date [34] has examined the fit of the patient experience of CrF with the concepts from the SRM. In that study, the majority of patient statements were classified as mental representations of fatigue, with fewer references to coping and appraisal. The authors concluded that the SRM was a valid organizing framework for CrF in patients undergoing treatment [34]. However, the experience of fatigue is likely to be different when the patient transitions into long-term survivorship [35]. The utility of the SRM at this later stage awaits investigation. Reviews of the literature have not reported on a trial that has used the SRM as a theoretical framework underpinning research into CrF in cancer survivors [36, 37]. Pertl et al. [14] carried out a thematic discourse analysis on the ‘additional comments’ left by 73 fatigued cancer patients and survivors as part of a questionnaire study on CrF. However, it may be the case that the questionnaire primed comments on related topics. Further, less than half the participants provided comments [14]. In order to build on these findings, this study used focus groups in order to allow participants to discuss the experience in more depth and with others who experience similar symptoms.

Accordingly, this qualitative study examines if the SRM is a useful framework to conceptualise CrF in long-term cancer survivors. In turn, this model may prove to be a useful integrated theoretical model for developing, evaluating and explaining the underlying mechanisms involved in CBT interventions for CrF. The use of such a model could enhance our understanding of the complex processes involved in the development and maintenance of CrF in some individuals.

## Methods

### Procedure

In line with Medical Research Council (MRC) guidelines [38], this study sought to identify if SRM theory could potentially be relevant and useful in the design of a psychological intervention for fatigue in post-treatment cancer survivors.

A contextualist approach was adopted to acknowledge the meanings applied to, and reality of, the experience of CrF, and to understand how the broader social context impacts on those meanings [39].

Focus groups were used as they enable discussion about the subjective experience of persistent fatigue, and they facilitate conversation about a topic that is not often addressed [40]. The study protocol was approved by the Institutional University Research Ethics Committee of the National University of Ireland Galway, Ireland. Details of the research procedure can be seen in Table 1.

**Table 1:** Consolidated criteria for reporting qualitative research (COREQ) checklist [76] for focus groups

Item	Description
Domain 1: Research team and reflexivity	
Personal Characteristics	
1. Interviewer/facilitator	Two authors (TC and BMG) conducted the focus groups
2. Credentials	TC: BA, MSc
	BMG: BA, MClinPsych, DipCrim, DipHealthSc, PhD, AFPsSI, Reg Psychol (PsSI), AFBPsS, CPsychol
3. Occupation	TC: PhD candidate
	BMG: Research Leader and Clinical Psychologist
4. Gender	TC: female
	BMG: male
5. Experience and training	TC: trained in qualitative research methods and design; experience in conducting focus groups
	BMG: trained in qualitative research methods and design; experience in facilitating clinical groups
Relationship with participants	
6. Relationship established	Participants contacted TC via email or telephone to discuss arrangements for the focus groups. Otherwise participants had no relationship with researchers
7. Participant knowledge of the interviewer	Participants were informed that the researcher was conducting a PhD in the area of cancer related fatigue and that her goal was to understand the symptom better by discussing it with people who lived with it.
8. Interviewer characteristics	Qualitative researcher and supervisor were both closely engaged in the research process and were therefore unable to completely avoid personal bias. This research sought to inform the content of an intervention.
Domain 2: study design	
Theoretical framework	
9. Methodological orientation and Theory	Thematic analysis was used in this study. A contextualist approach was adopted to acknowledge the meanings applied to, and reality of, the experience of CrF, and to understand how the broader social context impacts on those meanings [39].
Participant selection	
10. Sampling	Cancer survivors who self-reported ‘significant fatigue or reduced energy’ were eligible to take part. Self-selected Irish cancer survivors from the general public participated in this research.
11. Method of approach	From February to June 2014, cancer support groups and associations in the region were contacted. A press release was distributed to local media groups advertising the study.
12. Sample size	There were 18 participants in the study. There were four individuals in each of the first three focus groups and the final group had six attendees.
13. Non-participation	All participants who agreed on a date and time to attend took part in the focus groups.
Setting	
14. Setting of data collection	Data was collected in a meeting room in the School of Psychology at the University where the researcher is based.
15. Presence of non-participants	No one else was present besides the participants and researchers.
16. Description of sample	Demographic data can be seen in Table 3.
Data collection	
17. Interview guide	Questions based on a study by Barsevik et al [34] were utilised. These open-ended questions were posed to each of the groups: (a) what is your experience of fatigue? (b) What does the experience of fatigue mean to you? and (c) what do you do about your fatigue? These were the primary questions asked, with other topics being addressed as the conversation developed. If an opportune moment arose, other questions from Barsevik et al [34] were also included: (a) are there different types of fatigue? (b) How do other symptoms affect fatigue? and (c) what do you and/or your doctors and nurses recommend to manage fatigue?
18. Repeat interviews	No repeat interviews were carried out.
19. Audio/visual recording	Audio recording was used to collect the data.
20. Field notes	Field notes were made during and after the focus group.
21. Duration	Each of the focus groups was approximately 90 min in duration.
22. Data saturation	The researchers decided that data saturation had been achieved after the fourth focus group. The transcripts were reviewed as soon as possible after each interview. Saturation was achieved as no further additional new information began to emerge. It was agreed that the addition of new codes was unlikely after the fourth focus group [77].
23. Transcripts returned	Transcripts were not returned to participants for comment and/or correction.
Domain 3: analysis and findings	
Data analysis	
24. Number of data coders	Two data coders (TC and AMG) coded the data
25. Description of the coding tree	Coding Tree can be seen in Fig. 1.
26. Derivation of themes	Themes were identified in advance based on theory. The identified themes are reflective of patterns in the data and aim to provide a unified picture. Two researchers agreed on a clearly specified thematic coding manual which guided the interpretation of the data.
27. Software	Data was managed by hand
28. Participant checking	Participants did not provide feedback on the findings.
Reporting	
29. Quotations presented	Participant quotations were presented to illustrate the themes/findings. Each quotation identified using the participants’ age, gender, and cancer diagnosis.
30. Data and findings consistent	There is consistency between the data presented and the findings. The unit of analyses was the theme rather than the prevalence or frequency of statements. Some statements of quantification are included (e.g., statements such as often, sometimes), but do not always aim to provide estimates of prevalence.
31. Clarity of major themes	Codes identified in the open coding stage were discussed by two study authors until consensus was reached. A coding manual was developed (See Table 4 in Appendix 1) to clarify and define each of the themes. In stage two, the codes were checked in relation to pre-defined themes based on SRM. All major themes clearly presented in the findings.
32. Clarity of minor themes	There is a description of minor themes in the findings.

An interview script was designed in line with the questions asked by Barsevik et al [34] (See Appendix 2).

Thematic analysis was employed to identify, analyse and report themes within the data [42]. Further details of this process can be seen in Table 2.

**Table 2:** Process of data analysis

**Fig. 1:**
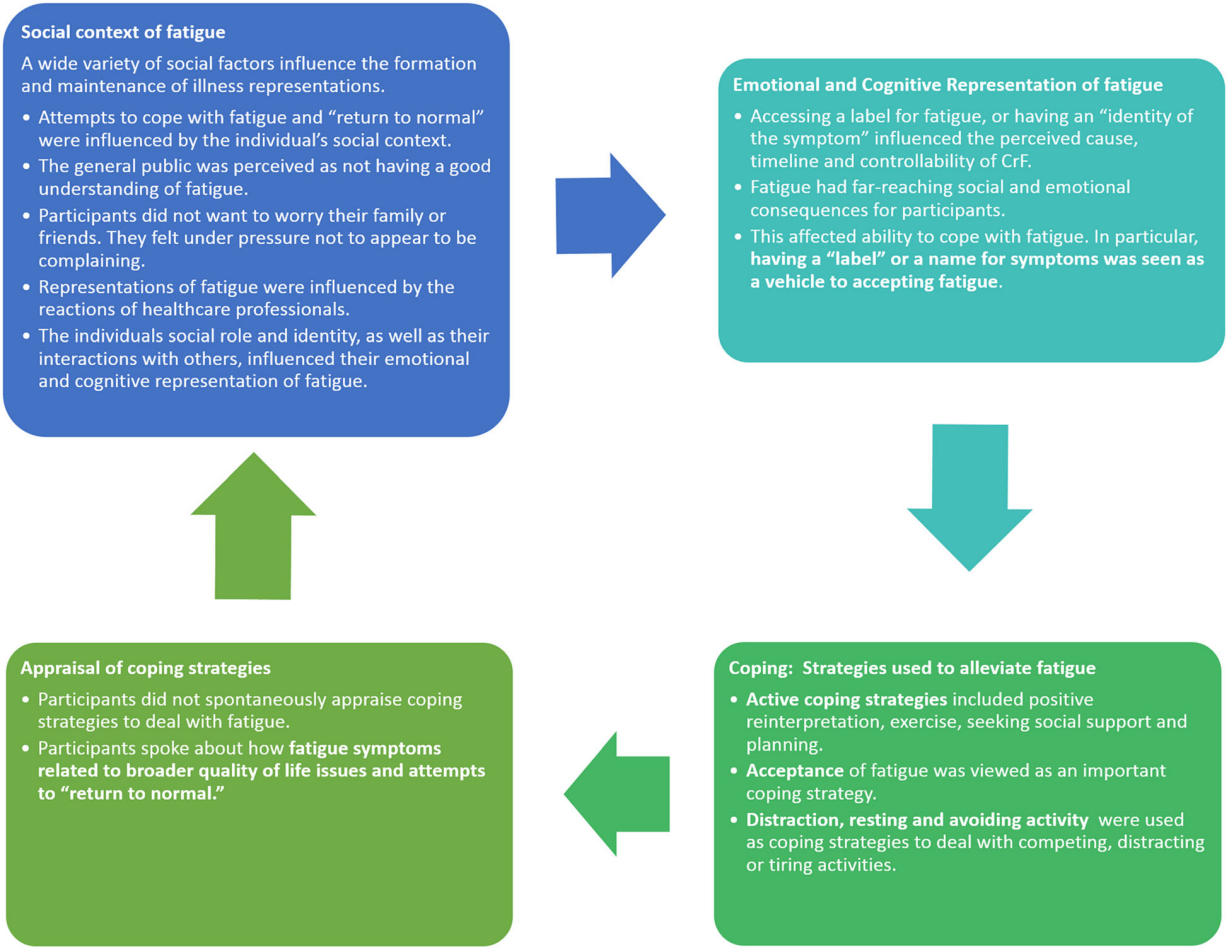
“A Self-Regulation Model of Cancer-Related Fatigue”

1. Coding was initially data-driven using an inductive approach to ensure that the data was analysed comprehensively, without trying to fit it into a pre-existing model or analytic preconceptions (Braun and Clarke, 2006 [39]). Two researchers (TC and AMG) processed initial features of the data that were of interest (codes). Each transcript was analysed separately and emerging codes were compared across groups. Discrepancies were discussed with co-authors (JW and BMG) until consensus was reached.
2. At the next stage of data analysis there was a shift towards the broader level of themes. Themes were items that represented some level of patterned meaning within the data (Braun & Clarke, 2006 [39]). Codes were organised using a theoretical thematic analysis, driven by SRM theory [41]. The analysis of the codes was theory-driven in order to address the specific research question, “Do participants’ subjective accounts fit with the components of the SRM?”
3. As themes were refined, the data set was reviewed to ensure that selected themes ‘worked’ and to identify any data that may have been previously overlooked. A thematic map of the data was produced (See Fig. 1).

The study was reported using the consolidated criteria for reporting qualitative research (COREQ) checklist for focus groups to ensure rigor in reporting in how the study was conducted.

Other features of the research that ensured validity [43] included:

- Clear statement of the aims of the research
- Justification of the methodology and research design used
- Ethical approval received for study
- Use of an interview schedule that was established a priori
- Rigor in data analysis achieved by following pre-defined steps of how to conduct thematic analysis
- Use of a coding manual for consistent analyses
- Use of interrater- coding in the data analyses

### Participants

Irish cancer survivors who self-reported ‘significant fatigue or reduced energy’ were eligible to take part. All participants were Caucasian. Four focus groups were held with 18 participants (Mean age 59.83, SD = 10.34). (See Table 3). Smaller groups were selected as- given the somewhat sensitive nature of the topic- the researchers felt that it may be difficult to get meaningful interaction among the participants in a larger group. The smaller number of participants, therefore, allowed for greater in-depth discussion in this exploratory research. Analysis of these discussions is based on themes that were reflective of patterns in the overall data rather than a reporting of the proportion of participants or groups expressing a theme. Participants gave consent to take part in a discussion about their fatigue and were informed that the sessions would be recorded.

**Table 3:** Demographic information for each of the participants

Gender	Age- Range	Cancer Type	Treatment Type				Time since treatment (months)
			Chemotherapy	Radiotherapy	Surgery	Other	
Male	66–80	Prostate		x		Hormonal therapy	36
Male	66–80	Prostate		x		Decopeptyl injections	7
Male	66–80	Prostate				Brachytherapy	60
Female	56–65	Breast		x			18
Female	66–80	Breast	x		x		42
Female	56–65	Breast		x	x		36
Female	66–80	Breast Stage III ductal	x	x	x		72
Female	40–55	Breast	x	x			18
Female	56–65	Breast	x	x	x		9
Female	40–55	Breast	x		x		24
Female	56–65	Triple Negative Breast	x	x	x		36
Male	66–80	Bowel and liver	x		x		18
Female	56–65	Stomach	x	x	x		72
Male	66–80	Stomach			x		24
Male	40–55	Testicular	x		x		48
Male	56–65	Rectum	x	x	x		26
Female	40–55	Non-Hodgkin Lymphoma	x	x			72
Male	40–55	Lymphoma	x	x			7

## Results

Participants reported that they valued the opportunity to discuss their experience with post-treatment CrF and enjoyed comparing their experiences with similar others. Individuals spoke freely, with little need for prompting from the facilitator. Details of the analyses and coding process can be seen in the coding manual (Appendix 1.) Analyses identified major themes that could be understood within the processes of the SRM: representation of symptoms; coping; appraisal of coping. The impact of the wider social context in the individual’s representation of their CrF was an overarching theme. These themes are described in further detail below and are illustrated in Fig. 1.

### Social context

The social context of fatigue was discussed. Participants were concerned that their symptoms were a deviation from the norm. The general public was perceived as not having a good understanding of fatigue. One participant (age 53, 36 months post-treatment) asserted that, “*It’s more socially acceptable in society to say ‘I’m a cancer patient’, than ‘I suffer from fatigue’.”*

### Social identity

The participants described the experience of CrF within the context of their own complex lives [44]. Participants did not want to worry their family or friends, and felt under pressure not to appear to be complaining. One woman (age 44, 72 months post-treatment) said “*you’re trying to explain to them that you’re tired and you’re flat and you just have no interest. Sometimes it’s hard to articulate.”* Individuals reported frustration at platitudes offered by others. Failure of others to understand that CrF was different from ‘normal’ fatigue, aging, or being busy was a common problem. One man (age 58, 26 months post-treatment) said, “*My friends and colleagues said “Look, we’re all getting old. It’s not you and your cancer at all. It’s just your age.”* As with previous studies, the findings indicated that understanding and support from others could be beneficial, but many often felt misunderstood and isolated when the reality of the “new normal” was not fully appreciated [44, 45]. Comparisons were likely if participants knew of others who had not experienced fatigue after cancer treatment. One woman (age 44, 72 months post-treatment) said that it “*makes you question it even more because you’re saying “why am I not like them?”* Meeting similar others helped participants to accept their own fatigue. One man (age 67, 7 months post-treatment) described how “*all of a sudden you realize that so many people have the same problem”.*

Participants discussed relationships and responsibilities. One woman (age 44, 72 months post-treatment) described pressure from her family to return to ‘normal’ saying “*It helps them cope as well. Because they see you as not being sick anymore and so they are indirectly pushing you to get back to your normal routine.”* One man (age 52, 7 months post-treatment) lamented the impact that CrF had had on his relationship with his son saying, “*I wouldn't be able to go playing ball with him. He’s gotten to where he doesn’t ask me. It’s a loss.”*

### Interaction with healthcare service

Perceptions of fatigue were impacted by the reactions of healthcare professionals to CrF. Participants felt that they were not adequately forewarned that fatigue would persist after treatment. Many said that fatigue *“was never mentioned.”* One person (age 53, 36 months post-treatment) noted that although fatigue was mentioned, *“nobody said 3 years down the line you’re still going to be nodding off.”*

Participants thought that doctors were dismissive when they mentioned fatigue. One woman (age 60, 18 months post-treatment) felt that her G.P. *“practically laughed”* when fatigue was mentioned. She believed that doctors were “*more into the treatment and you mention tiredness, they ignore you. It was swept under the carpet.”* Others agreed that healthcare providers were slow to engage in a conversation about fatigue. One man (age 52, 7 months post-treatment) said “*I feel like I’m telling her* [the doctor] *I’m tired all the time. She thinks I’m cuckoo.”* Another man (age 77, 36 months post-treatment) described how his doctors carried out various tests when he complained about fatigue, “*but not one of them has even acknowledged that it might be because I had cancer”.*

Positive experiences with healthcare professionals were also mentioned. One man (age 67, 7 months post-treatment) said he was “*lucky that I’ve a very good GP and he does listen. We do talk about fatigue.”* Many cited family members who worked in a healthcare profession as a support in terms of information provision and reassurance, especially in light of perceived gaps in care elsewhere.

Participants criticised a non-holistic approach to care, reporting that once physical causes of their fatigue were ruled out, their symptoms were often ignored. One participant (age 60, 18 months post-treatment) felt that healthcare professionals *“were very quick about curing things but they never worry about side-effects.”* Others considered fatigue *“a psychological aspect of the illness and the treatment that’s been left open ended”* (age 68, 42 months post-treatment). One man (age 67, 7 months post-treatment) said “*You’re left lonely. You’re left not having that support that you thought that the doctor might be able to give you.”*

Participants believed that ‘*quality of life*’ was an emerging concept in cancer care and influenced the recognition of side-effects such as fatigue. One man (age 77, 36 months post-treatment) suggested that “*maybe the reason the medical profession are in denial is that they don’t know how to cure it. They certainly don’t acknowledge it at all. It’s not within their competence to do anything about it so they just conveniently don’t recognise it.”*

### Cognitive and emotional representations of health threat

#### Identity

Participants voiced frustration that a label or definition of CrF was not provided to them. Acknowledgement of fatigue was viewed as a tool for acceptance that could reduce worry about the symptom and would justify the “*right to be tired”* (age 60, 18 months post-treatment). People emphasised the reality of fatigue, expressing the belief that fatigue was not something wholly psychological. One man (age 52, 7 months post-treatment) said *“it’s not imaginary. You really feel it. This exhaustion is true. It’s too strong.”* Another woman (age 53, 36 months post-treatment) echoed this sentiment saying “*there is not something wrong with our heads. It is actually real.”*

The lack of an available discourse for CrF influenced how people made sense of their symptoms, influencing the perceived cause, timeline, and controllability of CrF, in turn affecting their ability to cope with fatigue. One person (age 43, 24 months post-treatment) said, *“I was beginning to think that I was going silently crazy or something.”* Inadequate support was also raised as a potential causal factor in fatigue. One woman (age 60, 36 months post-treatment) believed that fatigue “*hits later down the line because you’re not in any cocoon. There is nobody there to look after you for the fatigue.*” Participants described having numerous medical tests that aimed to identify a physiological cause for the fatigue. These tests were often invasive and inconvenient. Importantly, they were also seen to reduce the validity of fatigue as an issue in and of itself, which led to feelings of confusion, uncertainty, and fear. Uncertainty about fatigue was also discussed in terms of the chronicity of fatigue. Participants wondered “*is this the way it’s going to be? Or will this ease?”* (Age 43, 24 months post-treatment). Some felt that they should be “*better by now*” (age 58, 26 months post-treatment). Doubt surrounding the prolonged experience of CrF led to emotional consequences. Many were uncertain if their fatigue was in line with their predicted time for recovery, with one saying *“I keep thinking this can’t be right. I cannot be this tired after three and a half years”* (age 60, 36 months post-treatment).

#### Cause

Fatigue was attributed to the culmination of stress during cancer. Others felt that they had taken on too much too soon after treatment, describing ‘*pressure on yourself to get back to your normal routine”* (age 60, 18 months post-treatment). Potential biological causes were also mentioned, including inflammation, endorphins, or cytokines. The role of cancer treatment in the development of symptoms was raised, with one noting “*The treatment made me tired. Treatment is worse than illness in some cases”* (age 67, 7 months post-treatment). A lack of concentration or ‘chemo-brain’ was also cited as a potential cause of fatigue. One participant (age 44, 72 months post-treatment) said “*You can’t focus and that- mentally- is kind of just adding to the tiredness.”*

Emotional responses, including fears of cancer recurrence, were also linked to the presence of fatigue. One woman worried that “*the cancer must be back. I think sometimes is there a bit of a bad cell left floating around in me that’s causing it?”* (Age 60, 36 months post-treatment).

Habits and mood were mentioned as possible causes of fatigue. One man (age 71, 18 months post-treatment) asked “*would tiredness be related to worry?”* Participants distinguished between fatigue and depression. Rather than attributing low mood to depressive symptoms, participants recognised low mood as being related by feeling “*so tired, you feel down because you’re not going to be able to do anything today”* (age 52, 7 months post-treatment)

#### Consequence

The impact of fatigue on quality of life and functional capacity was discussed, with one participant asking “*Without energy what can you have?”* (Age 55, 18 months post-treatment). Participants described learning to reconceptualise energy as a limited resource, as there was a ‘*cost*’ of activity and over-exertion. People avoided certain situations “*because it just won’t be worth it”* (age 44, 72 months post-treatment). One participant (age 53, 36 months post-treatment) said that “*Cancer was not the worst ordeal of my life. The diagnosis, the treatment, the surgery wasn’t debilitating- didn’t stop me from doing anything. But the fatigue does…”* The persistence of fatigue delayed return to a sense of ‘*normality*.’

Fatigue challenged the self-concept of many participants, with one noting *“it impacts on me not being able to be who I am”* (age 60, 36 months post-treatment). One woman (age 43, 24 months post-treatment) observed that *“you’re not able to do the things you want to do or you’re not where you want to be”.* Social lives were interrupted due to fatigue, resulting in *“a feeling of isolation” (*age 68, 42 months post-treatment). Some believed that fatigue was responsible for a lack of concentration and led to procrastination.

Fatigue had forced some individuals into early retirement, even when they *“had no intention of retiring just yet”* (age 67, 7 months post-treatment). One participant (age 44, 72 months post-treatment) said that she *“just could not face going back to work. I just really didn’t have the energy.”* One woman (age 60, 36 months post-treatment) described how retirement due to ill-health was “*part of a kind of a rejection”.*

Participants emphasised the “*difference between being tired and fatigue”* (age 53, 36 months post-treatment). One (age 60, 18 months post-treatment) spoke about how “*sleeping doesn’t seem to cure it,”* saying that “*every day for me was a groundhog day”.* Some reported heavy sleep at night. For others, getting to sleep posed a problem. One woman (age 68, 42 months post-treatment) stated that she had trouble “*staying asleep and then nodding off during the day.*

In terms of emotional representations, participants worried about fatigue, with one woman saying she found herself “*fretting to high heaven about it”* (age 60, 18 months post-treatment). Another described how “*thinking about it brings on anxiety*” (age 76, 24 months post-treatment). Distress as a consequence of CrF was addressed. One participant (age 57, 72 months post-treatment) pointed out that “*no matter how you try to be positive about it, it does get you down. It affects you mentally.”*

Participants felt guilty and ashamed about functional limitations, low energy levels, and pressure from others. One woman (age 68, 42 months post-treatment) regretted *“lost days”.* A mother in the group (age 44, 72 months post-treatment) said CrF affected her relationship with her children, feeling “*guilty because I didn’t do more things with them”.* Another participant (age 60, 36 months post-treatment) felt she was *“a failure”,* saying “*it’s not that I’m depressed. I’m frustrated. The only time I was ever angry about having cancer is the fact that it’s left me with fatigue.”* Another agreed that it was *“quite frustrating, debilitating in ways”* (age 43, 24 months post-treatment). A belief that participants ‘*should*’ be happy to have beaten cancer was also debated.

#### Timeline

Fatigue was described as having changed over time, and as being persistent. People described a “*dead tiredness, all the time”* (age 44, 72 months post-treatment) and how fatigue “*won’t go. It lingers”* (age 60, 36 months post-treatment)*.* Another explained, “*When you’re tired all the time it seems to drag on”* (Age 43, 24 months post-treatment).

The onset of fatigue during the day was addressed by participants. One (age 60, 18 months post-treatment) described fatigue *“creeping over me… gradually getting more and more tired. Until eventually you are flattened*.” Another experienced “*a weakness all over”* upon waking and felt *“very sluggish in the morning”* (age 43, 24 months post-treatment). Participants described a predictive pattern of fatigue, with one (age 67, 60 months post-treatment) saying “*you could nearly time yourself by it”.*

For certain individuals, fatigue symptoms had diminished over time. Some viewed fatigue as an acute symptom that would not go on forever. One (age 58, 26 months post-treatment) said “*I’m hoping it’ll go away. I thought I’d be ok this year but maybe next year.”*

#### Cure/control

Many participants believed that they had a lack of control over their tiredness. One (age 66, 72 months post-treatment) described her fatigue as “*totally uncontrollable”.* Another (age 52, 7 months post-treatment) mentioned that “*Sometimes you would just have to go to sleep.”* Conversely, others felt that they had gained control over their CrF, saying “*I think that it’s in your own hands and you have to plan and you have to work your way out of this lethargy*” (age 67, 7 months post-treatment).

### Coping: strategies used to alleviate fatigue

#### Active coping strategies

Positive reinterpretation was discussed as a means of staying optimistic with persistent fatigue. One woman (age 66, 72 months post-treatment) said it was necessary to *“be kinder to yourself. Be more forgiving.”* Some considered fatigue a small price to pay for surviving cancer. One (age 68, 42 months post-treatment) said that she was “*lucky to be here even though I feel wiped out”.*

Participants preferred not to give into their fatigue, with one participant (age 58, 26 months post-treatment) saying “*whatever I do, I won’t sleep during the day because if I sleep during the day I won’t sleep at night*.” Some used exercise to alleviate fatigue. One woman (age 53, 36 months post-treatment) said “*If you do get really tired, don’t sit. Go for a walk. Go out and do something. It can be the antidote.*” Another (age 57, 72 months post-treatment) said *“the more energy you use the more you get to replace it.”*

Participants sought advice, assistance, or information from doctors and support centres. For some, support received during cancer had extended into longer-term survivorship due to fatigue. One participant (age 59, 9 months post-treatment) noted “*I had my sister when I was sick to keep the house ticking over and she’s still there.”* Individuals sought out others for moral support, sympathy, or understanding. One participant felt *“it’s important to be out among people. You can’t be isolated”* (age 59, 9 months post-treatment)*.* This included support from other fatigued cancer survivors, with one man (age 67, 60 months post-treatment) concluding that “*we can learn from one another.”*

Planning helped participants to prepare coping strategies. One woman (age 44, 72 months post-treatment) said “*I just have to pace myself. You plan events. You have rest days before and afterwards. Make adjustments. You know you’re going to be flat.”*

#### Acceptance and emotion-focused coping

One described a process of learning *“to pace yourself better*” and to *“recognize your own limits”* (age 44, 72 months post-treatment). This was not always easy; one participant (age 60, 18 months post-treatment) said “*I don’t do things because I know I’m going to be tired. I know the consequence of it.”*

Acceptance of fatigue was difficult. One woman (age 60, 18 months post-treatment) voiced her frustration, saying *“who wants to listen to their body? You just want to go out and enjoy yourself.”* Another said that he “*just can’t accept it”* (age 52, 7 months post-treatment), whereas others felt it was important to acknowledge the reality of their situation. Another group member (age 60, 36 months post-treatment) reflected on how she felt she could cope better as a result of how she understood her symptoms, stating, “*I can manage my life if I know I’m going to be tired.”*

#### Distraction, resting and avoiding activity as coping strategies

Individuals often disengaged from activity to cope with fatigue. Napping was mentioned as a coping strategy, with one woman (age 60, 18 months post-treatment) saying that she sleeps “*on the couch every single evening”.* Research on CrF suggests that it may be better to avoid long or late afternoon naps as the combination of less daytime activity and more daytime sleep is associated with increased levels of CrF [36, 46, 47].

Mental disengagement was employed by participants to distract themselves from fatigue and the stress of dealing with it. One (age 43, 24 months post-treatment) said that she tries to “*forget about it and keep going*”, but in turn, described how she then feels more tired later as a consequence. The groups discussed the importance of recognising physical limitations rather than disengaging from them. Taking breaks and rest were discussed as useful. One woman (age 68, 42 months post-treatment) said “*Listen to your body. It’s saying slow down.”* Another (age 71, 18 months post-treatment) suggested “*if your body’s tired, just go away for 5 min and when you come back you’ll get through the day no problem.”* However, this was contested with one participant (age 60, 18 months post-treatment) saying, “*but when you raise your family… and you’re getting into your 60s and you want to go out and enjoy life… who wants to listen to their body? You just want to go out and enjoy yourself*.” One man (age 52, 7 months post-treatment), stated, *“Basically I don’t do things because I know I’m going to be tired. I just don’t do anything then because I know the consequence of it- that the recovery period is too long- and I say “sure it’s not worth it in the end”… so I don’t bother.”*

Participants felt it was sometimes best to avoid competing, distracting, or tiring activities. Learning to say “*no*” was recognised as an important skill for participants.

#### Appraisal of coping strategies

While participants were not asked explicitly to appraise the effectiveness of their coping, it was considered that certain questions could capture elements of this (“what do you do about your fatigue?” or “how do other symptoms affect fatigue?”)

In addition, in some instances the question “What have you found “works” for your fatigue” was included but these were not based on the Barsevik questions and arose naturally as the interviews progressed (See Appendix 2).

Within that context, the impact of the coping styles or strategies adopted was discussed. Participants discussed acceptance as a particularly important overall coping strategy. One woman (age 68, 42 months post-treatment) said “*I find I accept it now. It’s there. Deal with it. You either have a solution or you learn to live with it.”* Another (age 44, 72 months post-treatment) explained that *“you can reason with it better once you know that it’s got a name.”*

Some felt that their current coping strategies were ineffective. One woman (age 60, 18 months post-treatment) said “*I don’t do anything. I sit down and get very depressed. I’m not a very accepting person. I want to be like the way I was before it happened.*” Another (age 57, 72 months post-treatment) felt that her attitude prevented her from engaging in effective coping “*I go walking if I think I’m only going for a mile. Maybe I can’t manage it. But I couldn’t do it if I thought I was going for three miles. I don’t know is it my legs or my mind-set, you know? That’s the problem. Maybe my legs would take me the other two miles, but I can’t*.”

One man (age 52, 7 months post-treatment) described how “*some days you’d push yourself to do something and you knew you were tired but you’d achieve it. There is a great sense of achievement when you do something.”* Another (age 58, 26 months post-treatment) said “*I’m coping with it ok but I’m just finding time is long and I’d like to be back doing a full day’s work again but unfortunately just I’m not able for that yet.’* Participants identified challenges, with one woman (age 44, 72 months post-treatment) describing how initially she was "*so tired and so flat”,* and that “*it was only when the fog lifted”* that her family began to return to their *“own little routine and our own little life”.* In appraising her coping, she felt that “*it got easier once I recognized that you can’t do everything that you used to do.”*

These findings are depicted in Fig. 1.

## Discussion

These findings add to published quantitative and qualitative research to provide further understanding of the subjective experience of CrF in post-treatment cancer survivors. The SRM was developed to represent ‘lay’ understanding of illness experiences across all populations, yet to date very little research has applied the model to the experience of cancer survivors [32]. Findings in the current study, demonstrate that subjective experiences of post-treatment cancer survivors with CrF fit with the constructs of cognitive and emotional representations, coping strategies and appraisal as outlined in the SRM. This is in line with similar research conducted by Pertl et al (14) who conducted a thematic discourse analysis on the ‘additional comments’ of a questionnaire study on CrF in cancer survivors. Fatigue was understood as a part of the cancer experience that extended into longer-term survivorship. Without access to an available narrative to describe their experience, individuals engaged in a process of making sense of the fatigue themselves [14].

Participants emphasised the role of others, highlighting the influence of wider social discourse on their experience of fatigue. Dealing with social pressure to return to ‘normal’ after cancer was difficult for participants. Feelings of isolation associated with others’ lack of understanding regarding CrF might contribute to the symptom burden [48]. The social response to symptoms influenced participant representations of and coping with CrF, with some participants reporting hesitations in discussing their symptoms and feeling distressed or guilty as a consequence of CrF. Research has previously indicated that the social context is extremely pertinent to how representations are formed [49]. As with previous studies, the findings indicated that the social context of the “new normal” was crucial in how participants understood their symptoms. Reactions and support from others influenced these perceptions, and the coping response that followed [44, 45]. This serves to demonstrate the alignment of survivors’ views with the CrF. The individual’s coping with, and appraisal of, CrF can modify illness representations (i.e., in a feedback loop- See Fig. 1.) [30].

These qualitative results provide further insight into the specific ways that both the individuals’ perspective and the influence of social factors should be acknowledged in those with CrF. Individual perceptions and social context, as well as disease and treatment characteristics are important in planning survivorship care [50]. The SRM could be a useful framework for healthcare professionals to apply in assessments of survivor’s physical and psychosocial characteristics in order to effectively tailor care [51].

Many statements focused on the “identity” of CrF. A label was seen as a vehicle to accepting fatigue. In line with previous research, recognition of symptoms was considered crucial in learning to cope with fatigue and articulating the experience to others, including health professionals [52]. Barker et al [53] emphasised the significance of having a name for medically unexplained symptoms. The current study also showed that fatigue had far-reaching social and emotional consequences for participants. Many emotional consequences were associated with uncertainty that arose due to lack of recognition of CrF by others. Behavioural consequences such as the inconvenience of medical testing were also often linked to this uncertainty. Discussions relating to the timeline of CrF were similarly dominated by a sense of ambiguity. Likewise, individuals did not report a sense of control over their symptoms. Participants were not sure what to expect because CrF had already persisted longer than anticipated after the end of treatment. Others have also noted the link between cancer-related uncertainty and psychophysiological disruptions, highlighting a need to target this ambiguity in interventions for CrF (30). Factors such as comprehensibility, manageability, and meaningfulness can influence symptom perception. A sense of coherence may serve as a protective psychological factor in the adaptation process [54, 55].

The representations impacted on acceptance of fatigue and affected participants’ coping, as shown in previous research [56]. Perceived lack of support from family members and healthcare providers, as well as difficulties in trying to understand CrF also influenced participants’ ability to cope. Some participants described engaging in “active” coping strategies, such as exercise in managing CRF. Others reported taking naps or avoiding activity. Thus, participants may choose more maladaptive strategies for fatigue (e.g., daily/frequent napping) rather than recommended strategies (e.g., exercise [57]) depending on their representation of CrF. Beliefs about fatigue management influence coping strategies following cancer treatment [58].

Participants did not spontaneously appraise or reflect on the particular coping strategies they used to manage CrF. Evaluations focused more on general appraisals of attempts to regain a sense of ‘normality’ after treatment. Whitaker, Scott and Wardle [59] note that factors such as high external demands (e.g., family and work commitments) can impact interpretation of symptoms. Co-morbidities, stereotypes, and perceptions of aging can also bias appraisal of coping and expectations [37]. Acknowledgement of symptom seriousness from others may facilitate individual acceptance of fatigue and encourage appraisal of current coping strategies [59]. Current results add to those of a previous study which noted that appraisals of representations have been linked to seeking care in response to ambiguous [60] and prolonged symptoms [61]. Understanding how individuals appraise coping could help guide the development of tailored, proactive interventions to improve well-being [58].

Taken overall, this study suggests that the dynamic, self-regulatory structure of the SRM could serve as the basis for developing interventions for improving fatigue management [56]. This study extends on previous research that applied the SRM to patients’ experiences during cancer treatment [34, 62], providing insight into the unique representations of CrF in cancer survivors [63]. Persistent fatigue does not conform to generally accepted conceptualisations of survivorship, with treatment as an end-point of care [64].

The findings highlight a need for continued support to enhance quality of life after cancer treatment [35, 63]. Cancer itself and/or treatment initially trigger fatigue, but other factors may be responsible for persistence of CrF [17]. Exercise or psychosocial interventions are currently the treatment modalities of choice [65]. Existing interventions for CrF have focused on perpetuating factors such as beliefs and behaviours associated with fatigue, often using strategies based on cognitive behaviour therapy (CBT) [66]. CBT is a practical application of many of the theoretical constructs addressed in the SRM [67]. Very few randomized trials have explicitly applied the SRM to influence self-regulative symptom management [56]. None have explored how the SRM could be applied in interventions for CrF after cancer. SRM-based strategies could target how individuals think, feel and cope with their fatigue [56]. This study, therefore, concentrated solely on the fit of the discussions to the SRM rather than considering alternative models. Dempster et al [32] found that illness perceptions and coping play an important role in studies that link the SRM to wellbeing outcomes. It is still unclear how illness representations relate to coping, and how this might be applied in interventions (17). The current study suggests that interventions for CrF should be two-fold, targeting and measuring both the representation of symptoms and coping strategies [62]. A ‘top-down’ SRM approach to CrF would aim to create an overarching cognitive and emotional representation of fatigue as a manageable symptom, with coping and appraisal as targets for behaviour change [56]. In line with calls for improved methods of identifying and reporting the components of interventions, such an approach would need to assess the theoretical constructs of the SRM as proposed mechanisms of any change in fatigue symptoms [68].

### Limitations

Some limitations of this study should be acknowledged.

As the participants self-selected, this may have resulted in a biased sample not representing the wider population. Those frustrated with fatigue problems might have been more likely to self-nominate to participate in this research. Conversely, significantly fatigued people may have not felt well enough to participate. As this was an exploratory study of an under-researched symptom in Irish cancer survivors, it was decided that specific purposive sampling methods were not appropriate. This sampling method reflected the methods that would be used in any future intervention arising from the findings, and would give a sense of perceived need/interest in the topic from the community. However, the authors recognise that the current study may be susceptible to selection biases, in particular regarding the disproportionate number of breast cancer survivors who participated. For example, Wenger and Oliffe [69] suggest that men may be less comfortable discussing distressing situations or symptoms, and may feel more cautious about who they consider a safe source of support.

The collection of data via focus groups means that participants may conform to the perspectives of others in the group and findings may not reflect the views of individual participants [70]. Care was taken to ensure that each participant was given ample opportunity to express their perspective, however, some were naturally inclined to speak more than others. Researchers did make efforts to keep dominant respondents from taking over discussion.

The groups engaged in a free-flowing conversation with relatively little input from the researchers. However, issues raised by participants were likely to depend on their circumstances at the time of participation [71]. Although a variety of cancer types and treatments were represented, a more heterogeneous sample would broaden the content of the representations, coping strategies, and appraisals. Furthermore, information relating to education level was not gathered. Some evidence published prior to this research had suggested that factors such as ethnicity, educational attainment, and employment status were not associated with fatigue [72, 73]. However, recent evidence (published since the focus groups were conducted) suggests that lower education and pre-existing comorbidities may be associated with fatigue [74, 75].

Attempts were made to avoid bias in how the data was collected and interpreted. The moderators of the groups were experienced in qualitative research and followed an interview schedule. Questions were designed to be neutral and answerable. General questions were asked before specific questions.

While there were advantages to conducting qualitative rather than quantitative survey-based methods to learn more about CrF, some limitations must be considered. The analysis of these discussions is based on themes that were reflective of patterns in the overall data rather than a reporting of the proportion of participants or groups expressing a theme. Using qualitative methods did not provide information regarding the proportion or frequency of particular responses (See Table 1. Point 30).

The first author of this paper analysed the data and was also a moderator in the focus groups. This may have resulted in bias when reporting the findings. Experiences, beliefs, goals of the researcher and personality could bias analysis and reporting. However, efforts were made to minimise this bias by specifying the questions a priori in an interview schedule and following a coding manual. In addition, inter-rater reliability was used in the coding process.

## Conclusion

Overall, results indicated that post-treatment CrF can be described using concepts from the SRM. The findings contribute to the literature on the reconceptualization of cancer as a chronic illness by identifying perceived inadequacies in support available to those left with lingering side-effects after the completion of cancer treatment. The study demonstrated the complexity of the individuals’ meaning-making processes when the legitimacy of the health concern is contested. The current research identified specific elements of the SRM that were very pertinent for those with CrF. Participants felt inadequately prepared for persistent fatigue after cancer and were left confused, isolated and frustrated as a result. CrF should be approached as a complex psychosocial issue and considered from the patient perspective to facilitate better understanding and management of symptoms.

Prevailing models of healthcare promote patient-centered care for control of chronic symptoms and highlight the need for evidence based practice [38, 56]. Importantly, this study highlights how the SRM could be applied to CrF in post-treatment cancer survivors by providing a theoretical framework for understanding individuals’ representations, and coping strategies, and thus identifying targets for intervention [68].

## Appendix 1

**Table 4:** Code book

The SRM hypothesises that individuals create mental representations of their illness based on the concrete and abstract sources of information available to them in order to make sense of and manage the problem. It is the interpretation of this information that forms the first step in the process of seeking help, engaging in a coping strategy, or adopting an illness management regimen [78].	
*Social Context*	Abstract and concrete sources of information
	The perception and interpretation of the different sources of information influence other aspects of the participants’ perception of fatigue, including representations, coping with and appraisal of fatigue via symmetrical conceptual (abstract and prepositional) and schematic (concrete and perceptual) processes.
Label	Social Messages
Definition	The first source is information from the external social environment from perceived significant others. This refers to the general pool of ‘lay’ information already assimilated by the individual from previous social communication and cultural knowledge of the illness.
Description	• Deviation from norm • Source: Media/Similar others/family/friends • Type: vague/inaccurate/extensive/detailed Includes: how social response to the reports of their fatigue symptom is assimilated by the individual and in turn, impacts their acceptance and representation of the symptoms: • Reactions of and support offered by healthcare professionals • Reactions of and support offered by family and friends
Code2	
Label	Social Identity
Definition	This source of information relates to participants’ social role and identity. The expectations of others are included, as well issues that arise due to difficulties in articulating the experience of symptoms.
Description	• Expectations of others • Social comparisons (comparison to similar others) • Individuals role as parent/friend/employee
*Representation*	Health Threat (cognitive and emotional)
Code 4	
Label	Identity
Definition	
Description	• Label • Concrete signs/symptoms
Code 5	
Label	Cause
Definition	Dimension represents the beliefs regarding the factors that are responsible for causing the illness or disease.
Description	• Biological • Genetic • Psychological • Environmental • Emotion • Own behaviour
Code 6	
Label	Consequence
Definition	imagined and real refers to beliefs regarding the impact of the illness on overall quality of life or how it may affect functional capacity
Description	• Physical • Emotional • Social • Economic • Psychological
Code 7	
Label	Timeline
Definition	i.e., the time for the development of the disease, its duration, and time for recovery; Refers to the individual’s beliefs about the course of the illness (e.g., “My illness is chronic”) and time scale of illness symptoms (e.g., “The pain is persistent”).
Description	• Acute • Episodic • Cyclical • Chronic
Code 8	
Label	Cure/Control
Definition	Degree to which the disease can be prevented, cured, and kept from progressing.
	refers to the sensation of empowerment regarding performance of coping behaviours or the efficacy of treatment
Description	• limit/manage symptoms
*Coping*	
Code 9	
Label	Coping
Definition	Cognitive and behavioural actions we take (or do not take) to enhance health and to prevent, treat (i.e., cure or control), and rehabilitate from illness.
Description	• Distraction, Resting and Avoiding Activity • Problem-focused coping • Seeking social support. • Problem-focused coping–specific. As in Martin S. Hagger & Sheina Orbell (2003) A Meta-Analytic Review of the Common-Sense Model of Illness Representations, Psychology & Health, 18:2, 141–184, DOI: 10.1080/088704403100081321
*Appraisal*	
Code 11	Appraisal
Label	
Definition	Symptom and functional changes Evaluation of coping style/strategies adopted
Description	• What factors influenced coping? • Was my coping strategy effective?

## Appendix 2

### Focus Groups 2014 - Cancer-related fatigue

Interview schedule followed by interviewers during the focus groups. The aims of the interviews are outlined. The schedule also includes the semi-structured interview script that guided the focus group interviews.

The purpose of these focus group interviews is to discuss personal experiences of CRF from adult survivors of cancer. In particular, the study will aim to gather descriptions of the patients’ understanding of CRF and examine the fit of their descriptions to a theoretical model.

Does want to know:

1. How adults represent and understand their fatigue symptoms.
2. How adults cope with CRF
3. How adults appraise the impact of CRF.

Does not want to know:

1. how cancer *in general* has impacted their life
2. how they think others deal with or cope with fatigue

Goals of the research

General

- ➢ Develop a general understanding of target groups’ perceptions of CRF
- ➢ Identify the language and key concepts that the group uses to discuss CRF
- ➢ To frame the theoretical basis of the intervention. (i.e., To establish if Illness Perception theory could be applied in an Irish discussion of CRF)

Specific

- ➢ Key ideas that relate to the topic are identified
- ➢ The importance or significance of these key ideas can be described
- ➢ How strongly the participants feel about these key ideas can be identified
- ➢ Language and vocabulary are identified that relate to CRF and can be used in communication with participants.
- ➢ Solicit ideas in relation to the potential for an online intervention for CRF
- ➢ Questions and information from participants are available to assist with the further development of the research questions and purposes.
- ➢ Information from participants will verify hypothesis or help in refining hypotheses.

### Moderators’ guide: interview schedule

- Introduction
  - Welcome
    i. *“Welcome and thank you for coming to this focus group. Each of you has been invited to participate because your view is important to us. We know that you are very busy and we greatly appreciate your contribution to this project. This interview is not a test, nor should it in any way be viewed as a series of questions with right or wrong answers. Remember, we are very interested in what you think and feel. We want to know your opinions on these issues, and we are certainly not interested in your agreeing with the opinions and feelings of others. There may be times, however, when you do, and it is appropriate for you to let us know that as well.”*
    ii. *Here I have a participant information sheet for you to read. Once you’re happy that you’re clear on what is expected of you, you can sign our consent form. I can answer any questions you have.*
  - Purpose
    i. *“The purpose of this focus group interview is to discuss your personal experiences of cancer-related fatigue, as survivors of cancer.”*
  - Guidelines
    i. “*There are a few guidelines I would like to ask you to follow during the focus group interview. Frist, you do not need to speak in any particular order. When you have something to say, please do so.*
    ii. *Second, please do not speak while someone else is talking. Sometimes, the exchanges get emotional, and it is tempting to ‘jump in’ when someone is talking, but we ask you to refrain from doing so.*
    iii. *Third, remember that there are many people in the group and it is important that we obtain the point of view of each one of you. Fourth, you do not need to agree with what everyone or anyone in the group says, but you do need to state your point of view without making any negative comments or ‘put downs’.*
    iv. *Finally, because we have limited time together, I may need to stop you and to redirect our discussion. Does anyone have any questions? Ok, let’s begin*
- Warm-up
  - Set the tone
    i. *During the reception you had an opportunity to meet each other and to ask each other questions. To get the ball rolling, let’s start off with a brief introduction about yourself. Maybe tell us what type of cancer you had and how long it’s been since your treatment finished.*
  - Set participants at ease
- Clarification of terms
  - Establish the knowledge base of key terms through questions
  - Provide definitions of key terms
    i. *Just so that we’re all clear, I’m going to give you the standard textbook definition of cancer-related fatigue before we begin. It is defined as “a distressing persistent sense of tiredness or exhaustion related to cancer that is not proportional to recent activity and interferes with usual functioning”.*
    ii. *What is your experience of fatigue?*
- Establish Easy and non-threatening questions
  - The initial questions should be general and less threatening
    i. *What does the experience of fatigue mean to you*
    ii. *What do you do about your fatigue*
- Establish More difficult questions
  - The more difficult or personal questions should be determined
    i. *Are there different types of fatigue*
    ii. *How do other symptoms affect fatigue*
- Wrap-up
  - Identify and organize the major themes from the participant’s responses
    i. *Ok so from today it seems that we’ve covered quite a lot and heard some interesting points.*
  - Ensure that any conversational points not completed are mentioned
    i. *Unfortunately we didn’t have time to discuss….. further, but if you want to find out more you can contact some of the services on your Participant Information sheet*
- Member-Check
  - Determine how each member perceives selected issues
- Closing statements
  - Request anonymity of information
  - Answer any remaining questions
    i. *Has anyone any remaining questions about anything we’ve discussed today?*
  - Express thanks
    i. *Great! Thank you very much for coming today. Your help is greatly appreciated*

### Other questions

- What is fatigue
- Signs of fatigue
- What causes fatigue in cancer survivors…treatment? Or other causes such as pain, emotional distress, sleep problems, medications, nutrition, lack of exercise etc.?
- How to manage fatigue?
- How to cope with fatigue?
- How to find out more about fatigue? - Newspaper? Doctor? Online? Nurse? etc.?
- When did the fatigue first start?
- When did you notice that this fatigue is different from usual?
- Does anything make it better? Worse?
- Do you have any other problems or concerns?
- How has the fatigue affected the things you do every day?
- Do you use the internet to find out about symptoms?
- What do you and/or your doctors and nurses recommend to manage fatigue

## Abbreviations

CBT: Cognitive behaviour therapy
CrF: Cancer-related fatigue
MRC: Medical research council
SRM: Common-sense model of self-regulation of health and illness
